# Hospital costs associated with adverse events in people with diabetes in the UK

**DOI:** 10.1111/dom.14796

**Published:** 2022-06-29

**Authors:** Mi Jun Keng, Jose Leal, Louise Bowman, Jane Armitage, Borislava Mihaylova, ASCEND Study Collaborative Group

**Affiliations:** ^1^ Health Economics Research Centre, Nuffield Department of Population Health University of Oxford Oxford UK; ^2^ British Heart Foundation Centre of Research Excellence Oxford UK; ^3^ Clinical Trial Service Unit and Epidemiological Studies Unit, Nuffield Department of Population Health University of Oxford Oxford UK; ^4^ Medical Research Council Population Health Research Unit, Nuffield Department of Population Health University of Oxford Oxford UK; ^5^ Wolfson Institute of Population Health Queen Mary University of London London UK

**Keywords:** cardiovascular disease, cost‐effectiveness, diabetes complications, health economics

## Abstract

**Aim:**

To estimate the annual hospital costs associated with a range of adverse events for people with diabetes in the UK.

**Methods:**

Annual hospital costs (2019/2020) were derived from 15 436 ASCEND participants from 2005 to 2017 (120 420 person‐years). The annual hospital costs associated with cardiovascular events (myocardial infarction, coronary revascularization, transient ischaemic attack [TIA], ischaemic stroke, heart failure), bleeding (gastrointestinal [GI] bleed, intracranial haemorrhage, other major bleed), cancer (GI tract cancer, non‐GI tract cancer), end‐stage renal disease (ESRD), lower limb amputation and death (vascular, non‐vascular) were estimated using a generalized linear model following adjustment for participants' sociodemographic and clinical factors.

**Results:**

In the year of event, ESRD was associated with the largest increase in annual hospital cost (£20 954), followed by lower limb amputation (£17 887), intracranial haemorrhage (£12 080), GI tract cancer (£10 160), coronary revascularization (£8531 if urgent; £8302 if non‐urgent), heart failure (£8319), non‐GI tract cancer (£7409), ischaemic stroke (£7170), GI bleed (£5557), myocardial infarction (£4913), other major bleed (£3825) and TIA (£1523). In subsequent years, most adverse events were associated with lasting but smaller increases in hospital costs, except for ESRD, where the additional cost remained high (£20 090).

**Conclusions:**

Our study provides robust estimates of annual hospital costs associated with a range of adverse events in people with diabetes that can inform future cost‐effectiveness analyses of diabetes interventions. It also highlights the potential cost savings that could be derived from prevention of these costly complications.

## 1 INTRODUCTION

People with diabetes are at an increased risk of adverse events, ranging from cardiovascular to microvascular complications. These complications contribute to significant loss in life expectancy,^1^, ^2^ health‐related quality of life^3^, ^4^, ^5^ and are major cost drivers.^6^, ^7^, ^8^ It has been estimated that in the UK, people with diabetes incur an additional £3 billion in costs attributable to hospital use compared with people without diabetes.^9^ Given the growing health and economic burdens from the increasing prevalence of diabetes, there is a need to identify effective treatments and strategies to prevent and better manage diabetes to reduce the risks of these adverse events. Economic evaluations of these interventions require reliable estimates of healthcare costs associated with adverse events.

Robust estimation of the costs associated with adverse events requires accurate and contemporary patient‐level information on diabetes diagnosis and history, healthcare use and adverse event occurrences. There are only a limited number of studies in the UK that have reported the impact of diabetes complications on patient‐level costs.^10^, ^11^, ^12^, ^13^ The estimates by Alva et al.^12^ are frequently used in cost‐effectiveness analyses of diabetes treatments.^14^, ^15^, ^16^, ^17^, ^18^ However, these costs were derived using data from 1998 to 2007 and may not reflect the contemporary costs of diabetes complications because of changing patterns of care and the availability of new treatments. Contemporary estimates of costs reflecting modern treatment and management practices are needed to inform future economic analyses of diabetes treatments. A comparison between costs incurred by people with type 2 diabetes who experienced an incident cardiovascular event with those who did not was performed by McMeekin et al.^13^ using more recent data from the Scottish Care Information‐Diabetes Collaboration. The confidence intervals for estimates of secondary care costs associated with different cardiovascular events reported in the study were very wide, reflecting large uncertainty in their results. In this study, we use hospital data for participants in A Study of Cardiovascular Events in Diabetes (ASCEND) from 2005 to 2017 to estimate the impact of adverse events on annual hospital costs in the UK.

## 2 METHODS

### 2.1 Deriving annual costs of hospital use for the ASCEND study population

Details of the ASCEND study (ISRCTN60635500) have been reported previously.^19^, ^20^, ^21^ Briefly, ASCEND was a 2 × 2 factorial design trial that randomized 15 480 participants with established diabetes but without previous cardiovascular disease to 100 mg aspirin daily or matching placebo, and separately, to 1 g capsules daily containing omega‐3 fatty acids or matching placebo. Participants were recruited from 2005 to 2011, and were followed for an average of 7.4 years until 2017. Information on hospital attendances and admissions obtained from Hospital Episodes Statistics, Scottish Morbidity Records and Scottish accident and emergency data are available for all participants (excluding those who moved abroad or withdrew from the trial) in the ASCEND study in England, Wales and Scotland. Linked hospital data were not available for participants in Northern Ireland so they were excluded from the analysis.

Hospital activity data on admitted patient care (elective and non‐elective), outpatient attendances and procedures, and accident and emergency attendances (A&E), were included in the analysis. Each hospital attendance and admission (referred to as a hospital episode or finished consultant episode) was assigned a Healthcare Resource Group (HRG) using the 2017/2018 reference cost grouper.^22^ HRGs are UK groups of diagnoses (International Classification of Diseases‐10) and procedures (OPCS) codes that use comparable levels of healthcare resources. The cost for each hospital episode was calculated using the 2017/2018 NHS reference costs^23^ inflated to 2019/2020 values using the NHS cost inflation index^24^ in line with the National Institute for Health and Clinical Excellence guidelines.^25^ Dialysis sessions for chronic kidney diseases, typically performed in an outpatient setting, are recorded separately in the National Renal Dataset, which we do not have access to. Hence, the costs of maintenance dialysis were estimated separately (see Section 1 in Appendix S1 for further details) and included in the analysis so as not to underestimate the cost associated with end‐stage renal disease (ESRD). Cancelled or postponed outpatient episodes were excluded from the analysis. Unattended outpatient episodes were included to account for the opportunity costs of a missed scheduled appointment. We excluded unattended outpatient episodes to reflect the actual cost incurred in a sensitivity analysis.

Annual costs were established by aggregating the costs incurred by each participant each year relative to their recruitment date in ASCEND. We restrict the analysis to costs incurred within the follow‐up period in the study.

### 2.2 Identifying adverse events

The adverse events of interest include first occurrences of the following, for cardiovascular disease: myocardial infarction (MI), urgent and non‐urgent coronary revascularization, transient ischaemic attack, ischaemic stroke and heart failure; for bleeding: intracranial haemorrhage, gastrointestinal (GI) bleeds and other major bleeds (excluding eye bleeds); for cancer: GI tract cancer and non‐GI tract cancer (excluding non‐melanoma skin cancer); for other diabetes complications: lower limb amputation and ESRD; and for deaths, vascular (excluding intracerebral haemorrhage) and non‐vascular deaths. These events were identified from adverse events reported within the ASCEND study and from hospital episode data. Further details are provided in Table S1.

### 2.3 Imputation of missing data

Missing categorical patient characteristics were imputed with the modal category value. Missing clinical risk factors were imputed by predictive mean matching using the method of multivariate imputation by chained equations, with all covariates and history of event occurrence included in the imputation model, and averaged across 40 imputed datasets. Further details on imputation of missing data can be found in Section 2 in Appendix S1.

### 2.4 Statistical methods

Typical of healthcare costs data, we observed a high proportion of zero‐cost observations (no hospital use in‐year) and highly skewed distribution for non‐zero costs (Figure S1). Thus, for modelling the annual cost associated with adverse events, in addition to single‐equation generalized linear models (GLMs), we considered two‐part models (logistic regression for the first part predicting the likelihood of hospital use, GLMs for the second part predicting cost conditional on hospital use). The selection of the most appropriate model was based on common specification tests,^26^ predictive performance and parsimony (further details in Section 2 in Appendix S1).

Candidate covariates were identified from the literature and in discussion with clinical experts. The following variables were included in the statistical model: time since occurrence of adverse events as a categorical variable (no history, event occurring in‐year, event occurring the previous year and event occurring 2 or more years ago), current age, sociodemographic characteristics, type of diabetes (1 or 2) and clinical risk factors measured at recruitment into the trial (see Table S2). The history of adverse event occurrence in the annual period was stratified into four levels to reflect the immediate and longer‐term impacts of the adverse event on annual hospital costs. The levels were combined if there was no evidence of temporal difference based on the Wald test (*P* ≥ .05). Natural cubic splines were fitted to test for and explore the shape of non‐linearity of current age. Stepwise selection of covariates was performed based on the likelihood ratio test, with *P* values of less than .1 and less than .05 used for inclusion and exclusion, respectively. An indicator for the proportion of year observed was included in the model to account for censoring. Clustered standard errors were used to account for correlation between observations of annual costs for each patient.

## 3 RESULTS

Of the 15 480 participants in ASCEND, 44 (0.3%) participants from Northern Ireland, for whom we did not have hospital episode data, were excluded from the analysis. The baseline characteristics of the remaining 15 436 participants in England, Wales and Scotland are summarized in Table 1 (a comparison between baseline participant characteristics before and after imputation can be found in Table S3). In detail, 14 528 (94.1%) participants included in the analysis had type 2 diabetes. At recruitment, participants' average age was 63 years and their median duration of diabetes was 7 years. Participants were followed up for an average of 7.4 years, with only 97 (0.6%) participants lost to follow‐up, having withdrawn or moved abroad. A total of 120 420 person‐years of observations were available for analysis, and hospital use was recorded in 66% of these annual periods.

**TABLE 1:** Baseline characteristics of 15 436 participants included in the analysis

	N (%), mean (SD) or median (IQR)
Diabetes type	
Type 1	908 (5.9%)
Type 2	14 528 (94.1%)
Sex	
Male	9650 (62.5%)
Female	5786 (37.5%)
Smoking status	
Current smoker	1274 (8.3%)
Former/never smoker	13 989 (90.6%)
Missing	173 (1.1%)
Race	
White	14 892 (96.5%)
Indian/Pakistani/Bangladeshi	183 (1.2%)
African/Caribbean	140 (0.9%)
Missing	221 (1.4%)
Townsend Index^a^	
Q1: <−2.42 (least deprived)	6674 (43.2%)
Q2: ≥−2.42, <−0.44	3783 (24.5%)
Q3: ≥−0.44, <1.79	2492 (16.1%)
Q4: ≥1.79, <4.75	1814 (11.8%)
Q5: ≥4.75 (most deprived)	635 (4.1%)
Missing	38 (0.2%)
Diabetic retinopathy	
Y	3017 (19.5%)
N	12 277 (79.5%)
Missing	142 (0.9%)
Age (y)	63.3 (9.2)
Diabetes duration (y)	7 (3‐13)
Missing	853 (5.5%)
Body mass index (kg/m^2^)	31.1 (6.5)
Missing	118 (0.8%)
HbA1c (IFCC mmol/mol)	54.8 (12.9)
Missing	5652 (36.6%)
HDL cholesterol (mmol/L)	1.27 (0.35)
Missing	5665 (36.7%)
Non‐HDL cholesterol (mmol/L)	2.90 (0.84)
Missing	5665 (36.7%)
Systolic blood pressure (mmHg)	136.1 (15.3)
Missing	4430 (28.7%)
Diastolic blood pressure (mmHg)	77.1 (9.5)
Missing	4436 (28.7%)
Urinary albumin/creatinine ratio (mg/mmol)^b^	
<3	8503 (55.1%)
≥3	1242 (8%)
Missing	5691 (36.9%)
eGFR (ml/min/1.73m^2^)^c^	
<45	405 (2.6%)
≥45, <60	863 (5.6%)
≥60, <90	4007 (26%)
≥90	4511 (29.2%)
Missing	5650 (36.6%)

*Note*: 9879 (64%) of participants included in analysis returned a usable blood/urine sample.

Abbreviations: eGFR, estimated glomerular filtration rate; HDL, high‐density lipoprotein; IFCC, International Federation of Clinical Chemistry.

^a^ According to quintiles of Townsend Index in 2011 UK population.

^b^ 2403 participants who had undetectable albumin levels were reclassified as having no albuminuria (urinary albumin/creatinine ratio <3).

^c^ Calculated from blood cystatin C concentration using the Chronic Kidney Disease Epidemiology Collaboration formula.

Over the follow‐up period, 67 706 inpatient episodes (0.57 per person‐year), 375 952 outpatient episodes (3.12 per person‐year) and 28 423 A&E episodes (0.24 per person‐year) were recorded (Table S4). On average, participants with type 1 diabetes experienced a greater number of hospital episodes each year than participants with type 2 diabetes, particularly for outpatient episodes. Participants with type 1 diabetes had twice as many ophthalmology consultations (0.72 vs. 0.35 per person‐year), four times as many diabetes medicine speciality consultations (1.31 vs. 0.30), three times as many nephrology consultations (0.14 vs. 0.05) and twice as many eye procedures performed (0.30 vs. 0.14) in the outpatient setting (Table S5). Although there is smaller contrast in inpatient episodes between type 1 diabetes and type 2 diabetes (0.69 vs. 0.57), we see a greater number of episodes in the renal (0.05 vs. 0.02) and diabetes medicine specialities (0.04 vs. 0.01) (Table S6).

### 3.1 Annual hospital cost associated with adverse events

**FIGURE 1:**
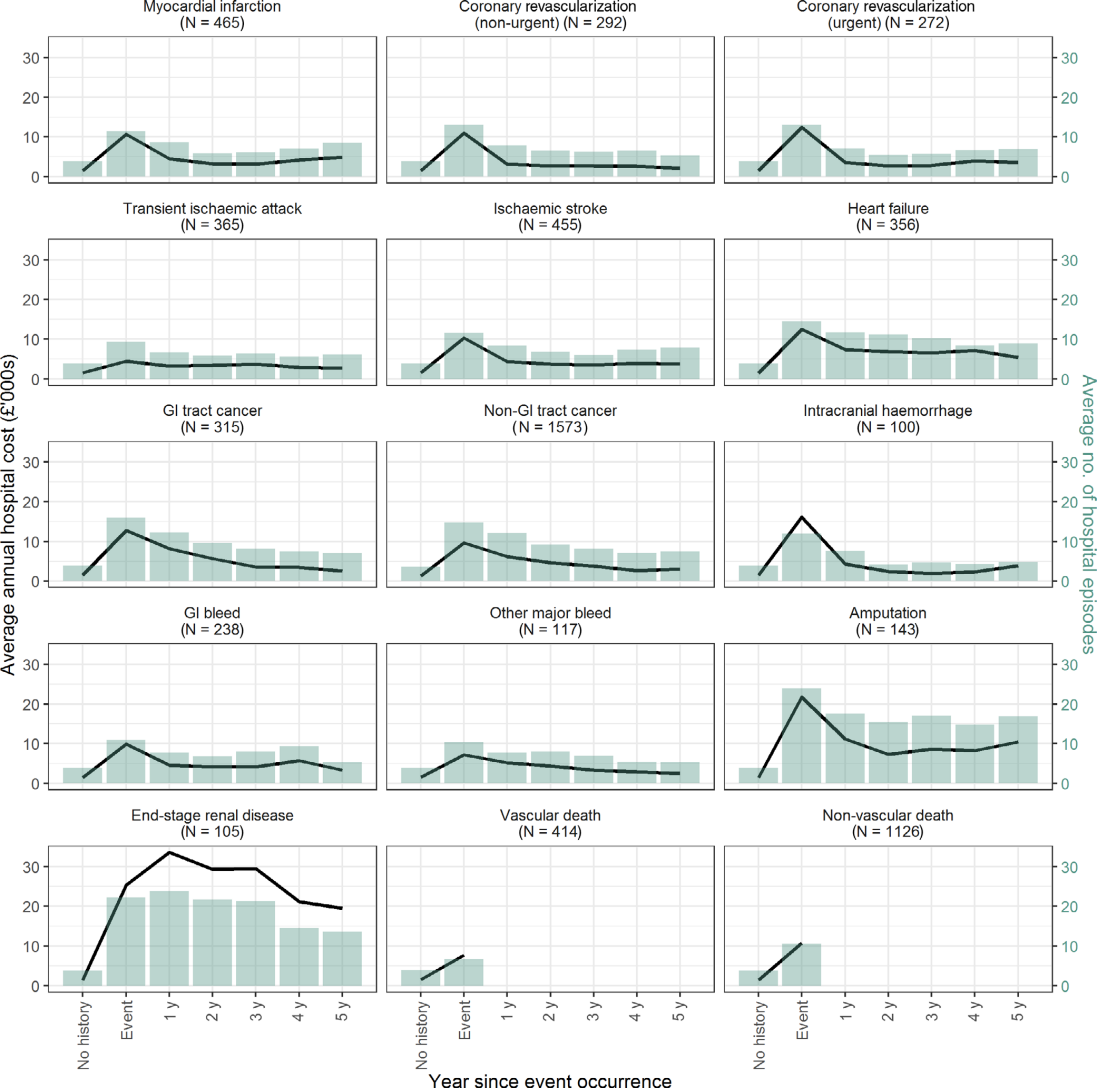
Average annual hospital cost and number of hospital episodes prior to, in year of, and in years subsequent to first adverse event occurrence. The black lines represent the average annual hospital cost and the coloured bars represent the average number of hospital episodes (hospital inpatient, outpatient and accident and emergency attendances and admissions) each year. Maintenance dialysis sessions are not included in counts of hospital episodes (see Appendix S1 for further details). The numbers in brackets are the number of participants who experienced each adverse event during the follow‐up of the ASCEND study. GI, gastrointestinal

Over the follow‐up period, 1573 (10.2%) participants had experienced a non‐GI tract cancer, 465 (3%) an MI, 455 (2.9%) an ischaemic stroke, 365 (2.4%) a transient ischaemic attack, 356 (2.3%) a heart failure, 315 (2%) a GI tract cancer, 292 (1.9%) a non‐urgent coronary revascularization, 272 (1.8%) an urgent coronary revascularization, 238 (1.5%) a GI bleed, 143 (0.9%) an amputation, 117 (0.8%) other major bleed, 105 (0.7%) an ESRD and 100 (0.6%) an intracranial haemorrhage. Further, 414 (2.7%) participants died of vascular causes, with 191 (1.2%) having had a vascular event in the same year; 1126 (7.3%) participants died of non‐vascular causes, with 363 (2.4%) having had a non‐vascular event in the same year. A peak in hospital use and hospital cost in the year of event occurrence was evident for all events (Figure 1, Table S7), and these tailed off immediately in the year following event occurrence for all adverse events except for ESRD, where cost remained high in the years after the ESRD event.

**FIGURE 2:**
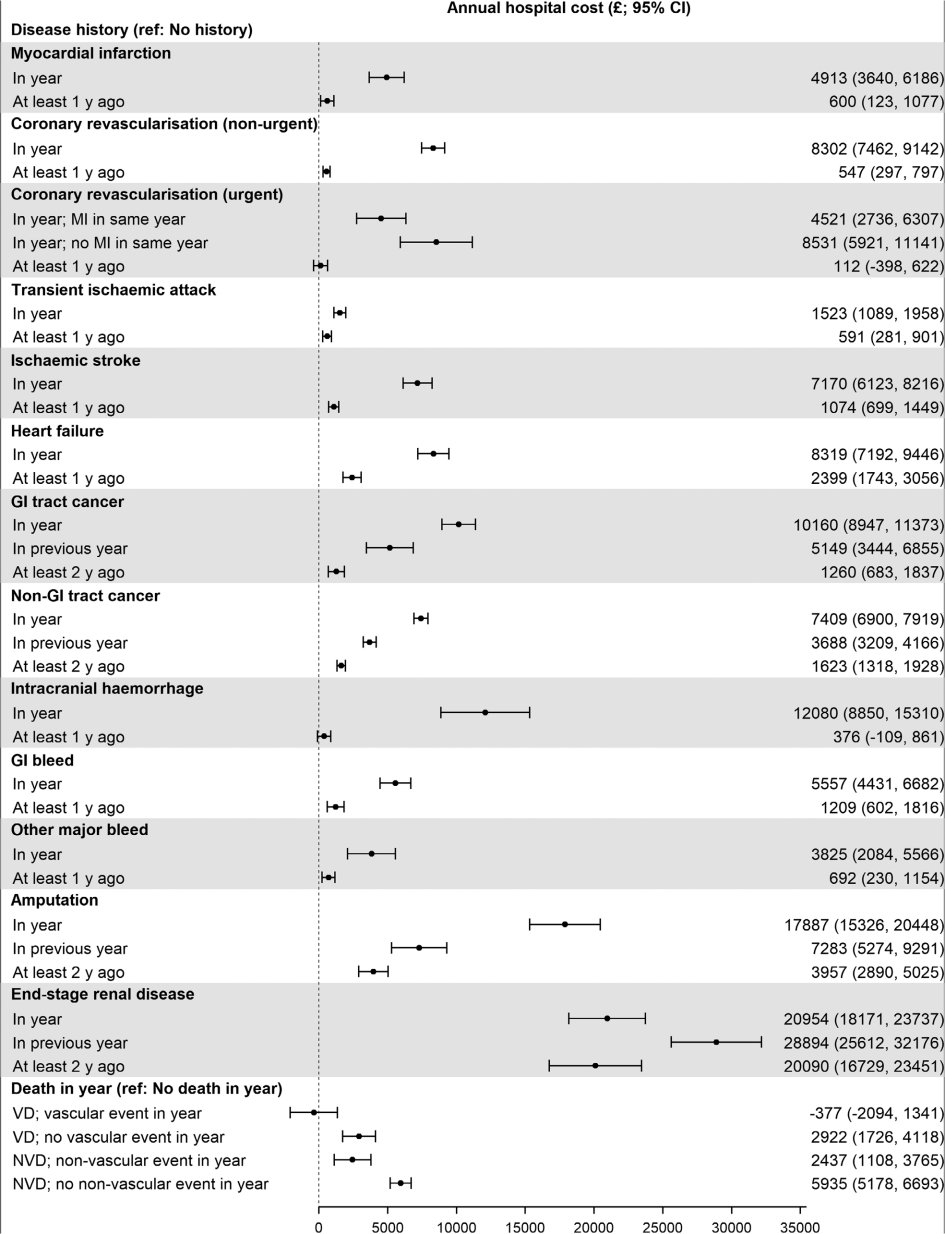
Annual hospital cost associated with adverse events. Vascular event refers to myocardial infarction (MI), coronary revascularization, transient ischaemic attack, ischaemic stroke or heart failure. Non‐vascular event refers to cancer, intracranial haemorrhage, gastrointestinal (GI) bleed, other major bleed, amputation or end‐stage renal disease. Estimation adjusted for diabetes type, sex, Townsend Index, age, body mass index, duration of diabetes, HbA1c, estimated glomerular filtration rate, presence of albuminuria, retinopathy and censoring. NVD, non‐vascular death; VD, vascular death

Results from the specification tests and a comparison of predictive performance of different model specifications can be found in Section 2 in Appendix S1. The single‐equation model (GLM with identity‐link function and Poisson variance) performed similarly to the two‐part model and was thus chosen for parsimony. The mean annual hospital cost incurred for a 60‐year‐old male with type 2 diabetes, living in a region in the lowest quintile of socioeconomic deprivation, with a body mass index (BMI) of less than 25 kg/m^2^, diabetes duration of less than 5 years, HbA1c less than 48 mmol/mol, estimated glomerular filtration rate (eGFR) of 90 ml/min/1.73m^2^ or higher, no albuminuria, no retinopathy and no history of adverse events, was £598 (95% CI: £538, £636). Relative to this reference case, the costs associated with adverse event occurrences, sociodemographic and clinical risk factors are presented in Table 2 and Figure 2.

**TABLE 2:** Annual hospital cost associated with sociodemographic characteristics, clinical risk factors and adverse events for people with diabetes: a multivariable generalized linear model

Variable	Excess annual hospital cost (£; 95% CI)
Diabetes type (ref: type 2)	
Type 1	149 (30, 267)*
Sex (ref: male)	
Female	104 (63, 146)**
Townsend Index (ref: Q1 [least deprived])	
Q2	8 (−17, 33)
Q3	4 (−47, 55)
Q4	170 (98, 242)**
Q5 (most deprived)	532 (357, 706)**
Age (per 10 y; centred at 60)	
Age	96 (71, 121)**
Age^2^	63 (46, 80)**
BMI (ref: <25 kg/m^2^)	
≥25, <30	13 (8, 18)**
≥30, <35	66 (23, 109)**
≥35	218 (158, 279)**
Diabetes duration (ref: <5 y)	
≥5, <10	36 (5, 67)*
≥10, <20	154 (100, 208)**
≥20	275 (184, 365)**
HbA1c (ref: <48 mmol/mol)	
≥48, <64	0 (−31, 31)
≥64	174 (107, 241)**
eGFR (ref: ≥90 ml/min/1.73m^2^)	
≥60, <90	32 (−16, 80)
≥45, <60	156 (49, 263)**
<45	651 (417, 886)**
Albuminuria (urinary albumin/creatinine ratio ≥ 3 mg/mmol) (ref: N)	
Y	190 (110, 269)**
Retinopathy (ref: N)	
Y	117 (62, 172)**
*Disease history (ref: no history)*	
MI	
In year	4913 (3640, 6186)**
At least 1 y ago	600 (123, 1077)*
Coronary revascularization (non‐urgent)	
In year	8302 (7462, 9142)**
At least 1 y ago	547 (297, 797)**
Coronary revascularization (urgent)	
In year	
MI in same year	4521 (2736, 6307)**
No MI in same year	8531 (5921, 11 141)**
At least 1 y ago	112 (−398, 622)
Transient ischaemic attack	
In year	1523 (1089, 1958)**
At least 1 y ago	591 (281, 901)**
Ischaemic stroke	
In year	7170 (6123, 8216)**
At least 1 y ago	1074 (699, 1449)**
Heart failure	
In year	8319 (7192, 9446)**
At least 1 y ago	2399 (1743, 3056)**
GI tract cancer	
In year	10 160 (8947, 11 373)**
In previous year	5149 (3444, 6855)**
At least 2 y ago	1260 (683, 1837)**
Other non‐GI tract cancer	
In year	7409 (6900, 7919)**
In previous year	3688 (3209, 4166)**
At least 2 y ago	1623 (1318, 1928)**
Intracranial haemorrhage	
In year	12 080 (8850, 15 310)**
At least 1 y ago	376 (−109, 861)
GI bleed	
In year	5557 (4431, 6682)**
At least 1 y ago	1209 (602, 1816)**
Other major bleed	
In year	3825 (2084, 5566)**
At least 1 y ago	692 (230, 1154)**
Amputation	
In year	17 887 (15 326, 20 448)**
In previous year	7283 (5274, 9291)**
At least 2 y ago	3957 (2890, 5025)**
End‐stage renal disease	
In year	20 954 (18 171, 23 737)**
In previous year	28 894 (25 612, 32 176)**
At least 2 y ago	20 090 (16 729, 23 451)**
*Death in year (ref: No death in year)*	
Vascular death	
Vascular event in year	−377 (−2094, 1341)
No vascular event in year	2922 (1726, 4118)**
Non‐vascular death	
Non‐vascular event in year	2437 (1108, 3765)**
No non‐vascular event in year	5935 (5178, 6693)**

*Note*: A generalized linear model with Poisson variance and identity‐link was used. The mean annual hospital cost incurred for the reference individual, who is a 60‐year‐old male with type 2 diabetes, living in the least deprived region, with BMI < 25 kg/m^2^, diabetes duration < 5 y, HbA1c < 48 mmol/mol, eGFR ≥ 90 ml/min/1.73m^2^, no albuminuria, no retinopathy and no disease history, is £598 (95% CI: £538, £636).

Vascular event refers to MI, coronary revascularization, transient ischaemic attack, ischaemic stroke or heart failure. Non‐vascular event refers to cancer, intracranial haemorrhage, GI bleed, other major bleed, amputation or end‐stage renal disease. Vascular death refers to any vascular‐related deaths that may or may not be a result of vascular events. Similarly, non‐vascular death refers to any non‐vascular–related deaths that may or may not be a result of non‐vascular events.

Abbreviations: BMI, body mass index; eGFR, estimated glomerular filtration rate; GI, gastrointestinal; MI, myocardial infarction. *P‐value < .05; **P‐value < .01.

Holding all else constant, in the year of an event, ESRD was associated with the largest increase in annual hospital cost of £20 954 (£18 171, £23 737), followed by amputation (£17 887 [£15 326, £20 448]), intracranial haemorrhage (£12 080 [£8850, £15 310]) and GI tract cancer (£10 160 [£8947, £11 373]). MI was associated with a cost of £4913 (£3640, £6186) and an additional £4521 (£2736, £6307) if urgent coronary revascularization was performed in the same year. We found no evidence of an additional cost for vascular death if vascular events (MI, coronary revascularization, transient ischaemic attack, ischaemic stroke, heart failure) occurred in the same year. When no vascular event occurred in the same year, vascular death was associated with an additional cost of £2922 (£1726, £4118). Non‐vascular death was associated with an additional cost of £2437 (£1108, £3765) if a non‐vascular event (cancer, intracranial haemorrhage, GI bleed, other major bleed, amputation, ESRD) occurred in the same year, and £5935 (£5178, £6693) otherwise.

There was evidence of a lasting impact on cost for all adverse events except urgent coronary revascularization and intracranial haemorrhage. Generally, the cost associated with an event declines significantly in subsequent years, holding all else constant. For example, the cost associated with GI tract cancer declines from £10 160 in the year of the event to £5149 (£3444, £6855) in the year after the event, and to £1260 (£683, £1837) in subsequent years. This was with the exception of ESRD, where the additional costs remained above £20 000 in years subsequent to the event (£28 894 [£25 612, £32 176] in the year after the event and £20 090 [£16 729, £23 451] in subsequent years).

### 3.2 Annual hospital costs associated with other factors

Holding all else constant and adjusting for the occurrence of adverse events, higher annual hospital costs were associated with having type 1 diabetes instead of type 2 diabetes, being female, living in a region of greater socioeconomic deprivation, having a higher BMI, a longer duration of diabetes, higher HbA1c, decreased renal function, and having retinopathy. Annual hospital costs also increased with age (Figure S2). Relative to a 60‐year‐old and holding all else constant, the annual hospital costs of a 70‐ and 80‐year‐old were higher by £159 and £444, respectively, following adjustments for other covariates.

Results were similar in the sensitivity analysis excluding 53 269 (12.4%) unattended outpatient episodes (Table S8).

## 4 DISCUSSION

We estimated the impact of cardiovascular, bleeding, cancer, lower limb amputation, ESRD and death events on annual hospital costs using an average of 7 years of data on more than 15 000 participants with diabetes from the ASCEND study. Holding all else constant, ESRD was associated with the largest acute impact on costs (£20 954), followed by amputation (£17 887), intracranial haemorrhage (£12 080) and GI tract cancer (£10 160). Vascular death with no vascular event in the same year was associated with additional costs of £2922. Non‐vascular death was associated with excess costs of £2437 if a non‐vascular event occurred in the same year and £5935 if not. Generally, we found that most adverse events were also associated with increased hospital costs in subsequent years for surviving participants.

ESRD was the largest cost driver both in‐year and in subsequent years of event occurrence. The costs associated with ESRD remained above £20 000 in years subsequent to event occurrence, largely driven by the costs of maintenance dialysis. Overall, 13 (12%) of 105 participants with ESRD had a transplant within the trial duration, with nine participants having a transplant within the first 2 years from date of first record of ESRD. The proportion of participants on dialysis among those with ESRD still being followed up decreased from 90% in the year after first record of ESRD to 74% after 4 years. This is as more participants received a transplant and possibly also because of better survival among transplant recipients.

Two other studies, conducted by Alva et al. and McMeekin et al.,^12^, ^13^ have previously analysed the impact of cardiovascular disease on costs in people with type 2 diabetes using patient‐level data in the UK context. McMeekin et al. found cerebrovascular disease to be associated with the largest cost, while Alva et al. found stroke to be associated with the second largest cost (after ischaemic heart disease [excluding MI]). Assuming approximately 80% of strokes are ischaemic,^27^ the combined impact of stroke on costs would be the largest among the cardiovascular events included in our study. Additionally, we found an impact of adverse events beyond the year of the event, as is reported in both of the other studies, as well as in several non‐UK–based studies.^28^, ^29^, ^30^, ^31^ Although we could draw some parallels in trends, comparison of the cost estimates themselves is less straightforward because of differences in populations, analytical approach and adverse event definitions. We found similar cost estimates to those reported in Alva et al. for stroke, lower estimates for MI and greater estimates for heart failure. We estimated the impact of coronary revascularizations on cost separately from MI, which could explain the discrepancy in the estimates for MI. The components of cost included in our study (hospital inpatient, outpatient and A&E records) were also different from those in Alva et al., which used patient‐reported resource use for non‐inpatient care. Additionally, the estimates of inpatient costs in Alva et al. were derived from hospital records from 1998 to 2007, but treatment patterns and costs have changed in the intervening years. Also, the costs of hospital episodes would have been captured more comprehensively in our study given the improvement in data quality of Hospital Episodes Statistics over time.^32^

Limited studies on costs associated with bleeding in the UK context are available,^33^, ^34^ and none specific to people with diabetes. Ramagopalan et al.^33^ found GI bleed to be associated with an excess cost of £3989 in the year of the bleed for patients with atrial fibrillation, which is smaller than what we found (£5557 [95% CI, £4431, £6682]). They found the excess cost to decrease to £1816 in the following year, while we report £1209 (£602, £1816) in all subsequent years following the event. Campbell et al.^34^ estimated the initial inpatient costs for acute GI bleeding to be £2458. However, this does not account for subsequent hospital episodes that may be attributable to the bleed, which were included in our estimate.

We found people with type 1 diabetes to incur £149 greater annual hospital costs than those with type 2 diabetes, holding all else constant. A previous study looking at costs of hospital use for diabetes patients in the UK found that people with type 1 diabetes had about 1.5 times more outpatient attendances than people with type 2 diabetes, which the authors suggested could be attributable to larger numbers of eye and foot checks and management of renal complications for those with type 1 diabetes.^9^ This agrees with observations in ASCEND, where participants with type 1 diabetes had a greater number of outpatient attendances than participants with type 2 diabetes (five vs. three per person‐year), with two to four times as many ophthalmology, diabetes medicine and nephrology consultations.

We found that people living in areas in the greatest sociodemographic deprivation quintile incur an additional £420 in hospital costs each year compared with those living in areas in the lowest sociodemographic deprivation quintile, even after adjusting for adverse event occurrences. Previous studies have found that socioeconomic gradients in healthcare costs were associated with increased morbidity.^35^, ^36^ Our finding suggests that there is excess morbidity among people living in the more deprived areas in addition to the clinical risk factors and adverse events included in the model.

Our study is not without limitations. First, we did not have ASCEND individual participant data from the National Renal Dataset and instead applied an estimated average annual dialysis cost based on proportions of patients on different dialysis modalities from the UK Renal Registry data,^37^ which has coverage of all renal centres in the UK. The variance in costs associated with ESRD may thus be underestimated. Second, we were unable to assess the excess costs associated with some common diabetes complications such as visual loss and hypoglycaemia as these events were not specifically sought in ASCEND. We were also unable to investigate further the effects of having multiple events. Other than MI and urgent coronary revascularization (n = 240), there were fewer than 35 participants who had other combinations of events co‐occurring in the same year, too few to reliably assess interactions between these events. Third, although no statistical difference was found between the estimates of cost associated with adverse events for type 1 diabetes and type 2 diabetes, this is probably attributable to the small number of participants with type 1 diabetes (6%) in our sample. Additionally, the ASCEND study population is largely of White ethnicity (97%), in contrast to 82% reported in a study of people with type 2 diabetes without cardiovascular disease identified from the general population in England.^38^ Most other sociodemographic and clinical risk factors are similar between the two cohorts, and these factors are adjusted for in our analysis. However, there may be unobservable factors associated with differences in hospital costs across ethnic groups, and we acknowledge this as a limitation. Fourth, an increase in uptake of modern diabetes medication, including glucagon‐like peptide‐1 receptor agonists (1.5% at baseline to 3.3% at approximately 6.5 years into follow‐up) and dipeptidyl peptidase‐4 inhibitors (2.2% to 8.2%), was observed in ASCEND (Table S9), and these drugs have been shown to reduce HbA1c and possibly reduce rehospitalization rates after a cardiovascular event.^39^ However, the diabetes care received by participants in ASCEND is expected to be similar to the general diabetes population during follow‐up. Lastly, our analysis is limited to costs of hospital care and does not include costs of primary care use or prescriptions. However, previous studies that have included these costs reported that the bulk of healthcare costs associated with complications for people with diabetes come from hospital use (more than 80% reported in McMeekin et al.).^8^, ^13^

Despite these limitations, the estimates in our study were derived from a large longitudinal patient‐level dataset with more than 120 000 person‐years of observations. This allowed us to reliably estimate the acute and long‐term impact on healthcare costs associated with adverse events. Our study also benefits from the high quality adverse events data reported in ASCEND. With the exception of heart failure, amputation and ESRD, all the other adverse events were clinically adjudicated. This made it possible, for example, to categorize strokes, which are usually suboptimally recorded in routinely collected health data.^40^ Our study is also the first to report costs associated with bleeding in people with diabetes in the UK. These estimates will be useful, for example, in evaluating net benefits of antiplatelet treatments in people with diabetes where cardiovascular benefits need to be weighed against bleeding risks. To aid the use of these cost estimates in cost‐effectiveness models, the covariance matrix of the regression coefficients is available at https://www.herc.ox.ac.uk/downloads/downloads-supporting-material-1.

Our study provides robust contemporary estimates of annual hospital costs associated with a range of adverse events in people with diabetes that can inform future cost‐effectiveness analyses of diabetes interventions. It also highlights the high costs associated with adverse events and the potential cost savings that could be derived from their prevention.

## AUTHOR CONTRIBUTIONS

MJK, JL and BM designed the study. MJK conducted the analyses and drafted the manuscript. JA and LB are principal investigators of the ASCEND study and provided important clinical insights in the development of the study and interpretation of results. All authors interpreted the results, critically revised and approved the final version of the manuscript. MJK is the guarantor of this work and, as such, had full access to all the data in the study and takes responsibility for the integrity of the data and the accuracy of the data analysis.

## CONFLICT OF INTEREST

All authors declare no conflict of interests relevant to this article.

### PEER REVIEW

The peer review history for this article is available at https://publons.com/publon/10.1111/dom.14796.

## Supporting information


**Appendix S1** Supporting Information

## Data Availability

The data that support the findings of this study are available from the Nuffield Department of Population Health, University of Oxford. Restrictions apply to the availability of these data, which were used under license for this study. Data are available at https://www.ndph.ox.ac.uk/data-access with the permission of the Nuffield Department of Population Health, University of Oxford.
