# Supplementary material for: Hospital costs associated with adverse events in people with diabetes in the UK
Source: Diabetes Obes Metab. 2022 Jun 29;24(11):2108–17. doi: 10.1111/dom.14796 (PMC9796307; doi:10.1111/dom.14796)
Supplement: Supplementary file 1 — Appendix S1 Supporting Information [file DOM-24-2108-s001.docx]

**Hospital costs associated with adverse events in people with diabetes in the UK**

**Supplemental materials**

Contents

[Supplementary Tables 2](#_Toc106870854)

[Table S1: Definitions and identification of adverse events of interest 2](#_Toc106870855)

[Table S2: Definitions of candidate variables included for selection 4](#_Toc106870856)

[Table S3: Baseline characteristics of 15436 participants included in the analysis, before and after imputation of missing data 6](#_Toc106870857)

[Table S4: Number and costs of different types of hospital episodes 9](#_Toc106870858)

[Table S5: Number and costs of outpatient hospital episodes by treatment specialty (for episodes with no procedure performed) and by HRG subchapter (for episodes with procedure performed) [top 20 occurrences] 10](#_Toc106870859)

[Table S6: Number and costs of inpatient hospital episodes by HRG subchapter [top 20 occurrences] 12](#_Toc106870860)

[Table S7: Average number of hospital episodes and hospital cost each year, stratified by time of event occurrence 13](#_Toc106870861)

[Table S8: Annual hospital cost associated with socio-demographic characteristics, clinical risk factors and adverse events for people with diabetes, excluding non-attended outpatient episodes (sensitivity analysis) 15](#_Toc106870862)

[Table S9: Self-reported diabetes medication use at baseline and at about 6.5 years into follow-up 16](#_Toc106870863)

[Supplementary Figures 17](#_Toc106870864)

[Figure S1: Distribution of non-zero annual hospital cost (£) 17](#_Toc106870865)

[Figure S2: Marginal effect of age on annual hospital cost 18](#_Toc106870866)

[Section S1: Costing dialysis 19](#_Toc106870867)

[Section S2: Further details on statistical methods 20](#_Toc106870868)

## Supplementary Tables

### Table S1: Definitions and identification of adverse events of interest

|  | Adverse events in trial | Hospital Episode Statistics data |
| --- | --- | --- |
| **Cardiovascular disease** |  |  |
| Myocardial infarction | ✓^†^  Evidence of cardiac necrosis and other evidence of acute MI and no other diagnosis was likely. Silent MI not included. |  |
| Coronary revascularisation (urgent) | ✓^†^  Coronary angioplasty, stenting or coronary artery bypass grafting. |  |
| Coronary revascularisation (non-urgent) | ✓^†^  Coronary angioplasty, stenting or coronary artery bypass grafting. |  |
| Transient ischaemic attack | ✓^†^  Transient episode of neurological dysfunction, lasting less than 24 hours, without clear evidence of acute infarction, haemorrhage, trauma or other cause. |  |
| Ischaemic stroke | ✓^†^  Acute symptomatic episode of focal or global neurological dysfunction as a result of infarction which lasted >24 hours, lead to death, or was associated with imaging evidence of acute infarct corresponding with clinical syndrome |  |
| Heart failure | ✓^‡^ | ✓ ICD-10 codes in primary diagnosis position:  I50 Heart failure; I110, I130, I132 Hypertensive heart disease with (congestive) heart failure; I255, I420, I425, I426, I427, I428, I429, I430, I431, I432, I438 Congestive cardiomyopathy |
| **Bleeding** |  |  |
| Intracranial haemorrhage | ✓^†^  Includes spontaneous intracranial bleeds or those associated with injury in the absence of major trauma |  |
| Gastrointestinal bleed | ✓^†^ |  |
| Other major bleed | ✓^†^  Major bleeds that required hospitalisation or transfusion, or was fatal or disabling. Excludes eye bleeds |  |
| **Cancer** |  |  |
| Gastrointestinal tract cancer | ✓^†^ |  |
| Non-gastrointestinal tract cancer | ✓^†^  Excludes non-melanoma skin cancer |  |
| **Other complications** |  |  |
| Amputation | ✓^‡^  Lower limb amputation | ✓ OPCS-4 codes: X09 Amputation of leg; X10 Amputation of foot; X11 Amputation of toe |
| End-stage renal disease | ✓^‡^  Includes participants who had dialysis or renal transplant | ✓ Algorithm & ICD-10 codes from Bush et al (2017)^1^ |
| **Death** |  |  |
| Vascular death | ✓^†^  Excludes death from intracerebral haemorrhage |  |
| Non-vascular death | ✓^†^ |  |

^†^Events corresponding to these endpoints were sought for and adjudicated in the ASCEND study.^2^ The date of event occurrence recorded in the study was used.

^‡^These endpoints were not sought for nor adjudicated in ASCEND. Hospital episode data was used to supplement the identification of these events. Earliest recorded date between the two sources used.

References:

1. Kathryn Bush, John Nolan, Qiuli Zhang, Will Herrington, Cathie Sudlow. Definitions of End Stage Renal Disease for UK Biobank Phase 1 Outcomes Adjudication. Published August 2017. Accessed May 7, 2021. <https://biobank.ndph.ox.ac.uk/ukb/ukb/docs/alg_outcome_esrd.pdf>
2. The ASCEND Study Collaborative Group. Effects of Aspirin for Primary Prevention in Persons with Diabetes Mellitus. *N Engl J Med*. 2018;379(16):1529-1539. doi:10.1056/NEJMoa1804988

### Table S2: Definitions of candidate variables included for selection

| Variables | Definitions or categories | Note |
| --- | --- | --- |
| Diabetes type | Type 1  Type 2 |  |
| Sex | Male  Female |  |
| Smoking status | Current smoker  Former/never smoker |  |
| Race | White  Indian/Pakistani/Bangladeshi  African/Caribbean |  |
| Townsend Index | Q1: <-2.42  Q2: >=-2.42, <-0.44  Q3: >=-0.44, <1.79  Q4: >= 1.79, <4.75  Q5: >= 4.75 | Measure of socioeconomic status calculated using postcode at randomisation. Higher value indicates greater deprivation. Stratified according quintiles of scores in 2011 UK population. |
| Hypertension | Y / N |  |
| Diabetic retinopathy | Y / N |  |
| Use of statin | Y / N |  |
| Use of ACE inhibitor or ARB | Y / N |  |
| Diabetes duration (years) | <10  >=10, <15  >=15, <25  >=25 |  |
| BMI (kg/m^2^) | <25  >=25, <30  >=30, <35  >=35 | Based on self-reported height and weight |
| HbA1c (IFCC mmol/mol) | < 48  >= 48, < 64  >= 64 | Conversion IFCC to DCCT unit:  48 mmol/mol = 6.5%;  64 mmol/mol = 8.0% |
| HDL cholesterol (mmol/L) | < 1.0  >= 1.0, < 1.5  >= 1.5 |  |
| Non-HDL cholesterol (mmol/L) | < 2.5  >= 2.5, < 3.5  >= 3.5 |  |
| Systolic blood pressure  (mmHg) | <130  >=130, <140  >=140 |  |
| Diastolic blood pressure  (mmHg) | <75  >=75, <85 |  |
| Urinary albumin/creatinine ratio (mg/mmol) | < 3  >=3 |  |
| eGFR (ml/min/1.73m^2^) | < 45  >= 45, < 60  >= 60, < 90  >=90 | Calculated from blood cystatin C concentration using the CKD-EPI formula |
| **Time-updated covariates** | | |
| Proportion of year observed | Range: 0 – 1 | Proportion of year participants were not lost to follow-up. Relevant for year in which participant was censored. For participants who were not censored in the year and for participants who died in year, proportion of year observed is 1. |
| Age (years) |  |  |
| Disease history  (adverse events listed below) | No history  In year  In previous year  At least 2 years ago | Time of event occurrence. |
| *Myocardial infarction* |  |  |
| *Coronary revascularization*  *(non-urgent)* |  |  |
| *Coronary revascularization*  *(urgent)* |  |  |
| *Transient ischaemic attack* |  |  |
| *Ischaemic stroke* |  |  |
| *Heart failure* |  |  |
| *GI tract cancer* |  |  |
| *Non-GI tract cancer* |  |  |
| *Intracranial haemorrhage* |  |  |
| *GI bleed* |  |  |
| *Other major bleed* |  | Other major bleed refers to major bleeding events that are not intracranial haemorrhage or GI bleed. |
| *Amputation* |  |  |
| *End-stage renal disease* |  |  |
| Death in year | No death in year  Vascular death (excludes death from intracerebral haemorrhage)  Non-vascular death |  |

BMI, body mass index; HbA1c, glycated haemoglobin; IFCC, International Federation of Clinical Chemistry; HDL, High-density lipoprotein; eGFR, estimated glomerular filtration rate; GI, gastrointestinal.

### Table S3: Baseline characteristics of 15436 participants included in the analysis, before and after imputation of missing data

|  | Before imputation | After imputation |
| --- | --- | --- |
| Region |  |  |
| England | 13960 (90.4%) | 13960 (90.4%) |
| Scotland | 439 (2.8%) | 439 (2.8%) |
| Wales | 1037 (6.7%) | 1037 (6.7%) |
| Diabetes type |  |  |
| Type 1 | 908 (5.9%) | 908 (5.9%) |
| Type 2 | 14528 (94.1%) | 14528 (94.1%) |
| Sex |  |  |
| Male | 9650 (62.5%) | 9650 (62.5%) |
| Female | 5786 (37.5%) | 5786 (37.5%) |
| Smoking status |  |  |
| Current smoker | 1274 (8.3%) | 1274 (8.3%) |
| Former/Never smoker | 13989 (90.6%) | 14162 (91.7%) |
| Missing | 173 (1.1%) | - |
| Race |  |  |
| White | 14892 (96.5%) | 15113 (97.9%) |
| Indian/Pakistani/Bangladeshi | 183 (1.2%) | 183 (1.2%) |
| African/Caribbean | 140 (0.9%) | 140 (0.9%) |
| Missing | 221 (1.4%) | - |
| Townsend Index^†^ |  |  |
| Q1: <-2.42 (least deprived) | 6674 (43.2%) | 6712 (43.5%) |
| Q2: >=-2.42, <-0.44 | 3783 (24.5%) | 3783 (24.5%) |
| Q3: >=-0.44, < 1.79 | 2492 (16.1%) | 2492 (16.1%) |
| Q4: >= 1.79, < 4.75 | 1814 (11.8%) | 1814 (11.8%) |
| Q5: >= 4.75 (most deprived) | 635 (4.1%) | 635 (4.1%) |
| Missing | 38 (0.2%) | - |
| Hypertension |  |  |
| Y | 9502 (61.6%) | 9614 (62.3%) |
| N | 5822 (37.7%) | 5822 (37.7%) |
| Missing | 112 (0.7%) | - |
| Diabetic retinopathy |  |  |
| Y | 3017 (19.5%) | 3017 (19.5%) |
| N | 12277 (79.5%) | 12419 (80.5%) |
| Missing | 142 (0.9%) | - |
| Prior statin use |  |  |
| Y | 11619 (75.3%) | 11619 (75.3%) |
| N | 3817 (24.7%) | 3817 (24.7%) |
| Prior use of ACE Inhibitor or ARB |  |  |
| Y | 9024 (58.5%) | 9024 (58.5%) |
| N | 6412 (41.5%) | 6412 (41.5%) |
| Age (years) |  |  |
| Mean (SD) | 63.3 (9.2) | 63.3 (9.2) |
| <50 | 1087 (7.0%) | 1087 (7.0%) |
| >=50, <60 | 4486 (29.1%) | 4486 (29.1%) |
| >=60, <70 | 6229 (40.4%) | 6229 (40.4%) |
| >=70, <80 | 3077 (19.9%) | 3077 (19.9%) |
| >=80 | 557 (3.6%) | 557 (3.6%) |
| Diabetes duration (years) |  |  |
| Mean (SD) | 9.8 (9.4) | 9.8 (9.3) |
| Median (IQR) | 7 (3 – 13) | 7 (4 – 13) |
| <5 | 4875 (31.6%) | 5017 (32.5%) |
| >=5, <10 | 4325 (28%) | 4810 (31.2%) |
| >=10, <20 | 3526 (22.8%) | 3700 (24%) |
| >=20 | 1857 (12%) | 1909 (12.4%) |
| Missing | 853 (5.5%) | - |
| Body-mass index (kg/m^2^) |  |  |
| Mean (SD) | 31.1 (6.5) | 31.1 (6.5) |
| <25 | 2064 (13.4%) | 2066 (13.4%) |
| >=25, <30 | 5455 (35.3%) | 5523 (35.8%) |
| >=30, <35 | 4510 (29.2%) | 4550 (29.5%) |
| >=35 | 3289 (21.3%) | 3297 (21.4%) |
| Missing | 118 (0.8%) | - |
| HbA1c (IFCC mmol/mol) |  |  |
| Mean (SD) | 54.8 (12.9) | 55.0 (11.2) |
| < 48 | 3261 (21.1%) | 4081 (26.4%) |
| >= 48, < 64 | 4651 (30.1%) | 8730 (56.6%) |
| >= 64 | 1872 (12.1%) | 2625 (17%) |
| Missing | 5652 (36.6%) | - |
| HDL cholesterol (mmol/L) |  |  |
| Mean (SD) | 1.27 (0.35) | 1.27 (0.29) |
| < 1.0 | 2162 (14%) | 2197 (14.2%) |
| >= 1.0, < 1.5 | 5506 (35.7%) | 10971 (71.1%) |
| >= 1.5 | 2103 (13.6%) | 2268 (14.7%) |
| Missing | 5665 (36.7%) | - |
| Non-HDL cholesterol (mmol/L) |  |  |
| Mean (SD) | 2.90 (0.84) | 2.90 (0.69) |
| < 2.5 | 3383 (21.9%) | 3731 (24.2%) |
| >= 2.5, < 3.5 | 4379 (28.4%) | 9595 (62.2%) |
| >= 3.5 | 2009 (13%) | 2110 (13.7%) |
| Missing | 5665 (36.7%) | - |
| Systolic blood pressure (mmHg) |  |  |
| Mean (SD) | 136.1 (15.3) | 135.9 (13.3) |
| <130 | 3389 (22%) | 4245 (27.5%) |
| >=130, <140 | 3080 (20%) | 5649 (36.6%) |
| >=140 | 4537 (29.4%) | 5542 (35.9%) |
| Missing | 4430 (28.7%) | - |
| Diastolic blood pressure (mmHg) |  |  |
| Mean (SD) | 77.1 (9.5) | 77.3 (8.3) |
| <75 | 4217 (27.3%) | 5266 (34.1%) |
| >=75, <85 | 4600 (29.8%) | 7772 (50.3%) |
| >=85 | 2183 (14.1%) | 2398 (15.5%) |
| Missing | 4436 (28.7%) | - |
| Urinary albumin/creatinine ratio (mg/mmol)^‡^ |  |  |
| < 3 | 8503 (55.1%) | 13505 (87.5%) |
| >= 3 | 1242 (8%) | 1931 (12.5%) |
| Missing | 5691 (36.9%) | - |
| eGFR (ml/min/1.73m^2^)^§^ |  |  |
| < 45 | 405 (2.6%) | 540 (3.5%) |
| >= 45, < 60 | 863 (5.6%) | 1345 (8.7%) |
| >= 60, < 90 | 4007 (26%) | 6007 (38.9%) |
| >= 90 | 4511 (29.2%) | 7544 (48.9%) |
| Missing | 5650 (36.6%) | - |

Summary values are N (%) unless otherwise specified. IQR, interquartile range; HbA1c, glycated haemoglobin; IFCC, International Federation of Clinical Chemistry; HDL, High-density lipoprotein; eGFR, estimated glomerular filtration rate. 9,879 (64%) of participants included in analysis returned usable blood/urine sample.

^†^ According to quintiles of Townsend index in 2011 UK population.

^‡^2403 participants who had undetectable albumin levels were reclassified as having no albuminuria (urinary albumin/creatinine ratio <3).

^§^Calculated from blood cystatin C concentration using the CKD-EPI formula.

### Table S4: Number and costs of different types of hospital episodes

|  | No. of episodes (%) | No. of episodes per person-year | Cost (£) per episode, median (IQR) |
| --- | --- | --- | --- |
| **All participants (N = 15,436; No. of person-years = 120,420)** | | | |
| Inpatient | 67,706 (14.3%) | 0.57 | 828 (476, 2074) |
| Outpatient | 375,952 (79.6%) | 3.12 | 126 (96, 147) |
| A&E | 28,423 (6.0%) | 0.24 | 180 (110, 219) |
| **Participants with type 2 diabetes (N = 14,528; No. of person-years = 112,492)** | | | |
| Inpatient | 62,720 (14.8%) | 0.56 | 848 (476, 2080) |
| Outpatient | 335,932 (79.1%) | 2.99 | 125 (96, 149) |
| A&E | 26,019 (6.1%) | 0.23 | 180 (110, 219) |
| **Participants with type 1 diabetes (N = 908; No. of person-years = 7928)** | | | |
| Inpatient | 4986 (10.5%) | 0.63 | 712 (422, 1856) |
| Outpatient | 40,020 (84.4%) | 5.05 | 134 (104, 142) |
| A&E | 2404 (5.1%) | 0.30 | 170 (110, 219) |

IQR, interquartile range. Maintenance dialysis sessions are not included in hospital episodes (see Section S1 for more information).

### Table S5: Number and costs of outpatient hospital episodes by treatment specialty (for episodes with no procedure performed) and by HRG subchapter (for episodes with procedure performed) [top 20 occurrences]

|  | | Cost (£) per episode,  median (IQR) | No. of episodes per 100 person-year | | |
| --- | --- | --- | --- | --- | --- |
| Treatment specialty / HRG subchapter | |  | All | Type 2 diabetes | Type 1 diabetes |
| **Episodes with no procedure performed, by treatment specialty** | | | | | |
| 130 | Ophthalmology | 92 (92, 92) | 37.1 | 34.6 | 72.1 |
| 307 | Diabetic Medicine | 142 (142, 142) | 36.9 | 30.2 | 130.9 |
| 110 | Trauma & Orthopaedics | 116 (116, 141) | 26.8 | 26.3 | 34.3 |
| 650 | Physiotherapy | 52 (52, 52) | 14.7 | 14.2 | 22.0 |
| 653 | Podiatry | 47 (47, 47) | 10.1 | 10.1 | 10.0 |
| 101 | Urology | 104 (104, 126) | 10.0 | 10.2 | 7.1 |
| 320 | Cardiology | 123 (123, 150) | 9.9 | 10.0 | 8.3 |
| 300 | General Medicine | 158 (158, 158) | 9.3 | 8.2 | 24.2 |
| 100 | General Surgery | 130 (130, 158) | 7.9 | 8.0 | 7.9 |
| 330 | Dermatology | 105 (105, 123) | 6.4 | 6.4 | 7.4 |
| 340 | Respiratory Medicine | 141 (141, 196) | 5.8 | 6.0 | 3.3 |
| 800 | Clinical Oncology (Previously Radiotherapy) | 129 (129, 129) | 5.5 | 5.6 | 4.2 |
| 361 | Nephrology | 174 (174, 174) | 5.3 | 4.7 | 14.0 |
| 301 | Gastroenterology | 142 (142, 179) | 5.3 | 5.3 | 5.6 |
| 410 | Rheumatology | 131 (131, 131) | 5.0 | 4.8 | 8.9 |
| 120 | ENT | 96 (96, 115) | 4.9 | 4.8 | 6.4 |
| 303 | Clinical Haematology | 154 (154, 154) | 4.9 | 5.1 | 2.4 |
| 302 | Endocrinology | 151 (151, 151) | 4.3 | 3.9 | 9.3 |
| 840 | Audiology | 87 (87, 87) | 3.9 | 4.1 | 2.1 |
| 370 | Medical Oncology | 161 (161, 161) | 3.7 | 3.7 | 3.4 |
| **Episodes with procedures performed, by HRG subchapter** | | | | | |
| BZ | Eyes and Periorbita Procedures and Disorders | 114 (114, 121) | 15.1 | 14.0 | 30.1 |
| JC | Skin Procedures | 151 (117, 155) | 6.7 | 6.6 | 7.8 |
| CA | Ear, Nose, Mouth, Throat and Neck Procedures | 105 (105, 125) | 4.0 | 4.0 | 4.5 |
| LB | Urological and Male Reproductive System Procedures and Disorders | 151 (151, 194) | 2.2 | 2.2 | 1.2 |
| SC | Radiotherapy | 154 (154, 211) | 2.1 | 2.2 | 1.3 |
| DZ | Respiratory System Procedures and Disorders | 181 (150, 195) | 1.3 | 1.4 | 1.0 |
| EY | Interventional Cardiology for Acquired Conditions | 141 (139, 141) | 1.2 | 1.2 | 0.7 |
| HN | Orthopaedic Non-Trauma Procedures | 154 (151, 172) | 0.8 | 0.8 | 1.5 |
| FE | Digestive System Endoscopic Procedures | 181 (177, 308) | 0.8 | 0.8 | 0.5 |
| MA | Female Reproductive System Procedures | 186 (184, 213) | 0.7 | 0.7 | 0.7 |
| SB | Chemotherapy | 692 (483, 1,147) | 0.7 | 0.7 | 0.4 |
| CD | Dental and Orthodontic Procedures | 172 (170, 201) | 0.6 | 0.6 | 0.3 |
| AB | Pain Management | 139 (125, 139) | 0.5 | 0.5 | 0.4 |
| RD | Diagnostic Imaging Procedures | 152 (124, 184) | 0.4 | 0.4 | 0.3 |
| AA | Nervous System Procedures and Disorders | 189 (189, 189) | 0.3 | 0.3 | 0.4 |
| YR | Vascular Imaging Interventions | 141 (141, 153) | 0.2 | 0.2 | 0.6 |
| FF | Digestive System Open and Laparoscopic Procedures | 171 (159, 208) | 0.2 | 0.2 | 0.1 |
| YJ | Breast Imaging Interventions | 289 (218, 340) | 0.2 | 0.2 | 0.3 |
| RN | Nuclear Medicine Procedures | 325 (278, 545) | 0.2 | 0.2 | 0.2 |
| LA | Renal Procedures and Disorders | 230 (203, 249) | 0.1 | 0.1 | 0.1 |

### Table S6: Number and costs of inpatient hospital episodes by HRG subchapter [top 20 occurrences]

| HRG subchapter | Cost (£) per episode,  median (IQR) | No. of episodes per 100 person-year | | |
| --- | --- | --- | --- | --- |
|  |  | All | Type 2 diabetes | Type 1 diabetes |
| FE Digestive System Endoscopic Procedures | 525 (476, 632) | 6.5 | 6.6 | 5.5 |
| BZ Eyes and Periorbita Procedures and Disorders | 900 (326, 900) | 5.1 | 4.9 | 7.2 |
| LB Urological and Male Reproductive System Procedures and Disorders | 749 (493, 1,853) | 3.6 | 3.7 | 2.0 |
| HN Orthopaedic Non-Trauma Procedures | 2,659 (1,288, 6,267) | 3.5 | 3.5 | 4.2 |
| DZ Respiratory System Procedures and Disorders | 1,472 (476, 2,228) | 3.5 | 3.6 | 2.2 |
| EB Cardiac Disorders | 559 (386, 1,949) | 3.2 | 3.3 | 2.7 |
| SB Chemotherapy | 1,000 (695, 1,660) | 2.8 | 2.8 | 2.6 |
| FD Digestive System Disorders | 1,330 (447, 2,100) | 2.6 | 2.6 | 2.9 |
| WH Poisoning, Toxic Effects, Special Examinations, Screening  and Other Healthcare Contacts | 437 (338, 617) | 2.6 | 2.5 | 3.9 |
| LA Renal Procedures and Disorders* | 460 (453, 2226) | 2.0 | 1.9 | 4.6 |
| AA Nervous System Procedures and Disorders | 1,397 (523, 3,473) | 2.0 | 1.9 | 3.4 |
| SA Haematological Procedures and Disorders | 499 (294, 527) | 1.8 | 1.8 | 1.7 |
| EY Interventional Cardiology for Acquired Conditions | 1,784 (1,053, 2,927) | 1.5 | 1.6 | 1.5 |
| HD Musculoskeletal and Rheumatological Disorders | 398 (355, 2,378) | 1.1 | 1.1 | 1.8 |
| JC Skin Procedures | 670 (670, 1,035) | 1.1 | 1.1 | 1.1 |
| KB Diabetic Medicine | 636 (428, 2,282) | 1.1 | 0.9 | 3.6 |
| FF Digestive System Open and Laparoscopic Procedures | 2,571 (1,369, 5,521) | 1.1 | 1.1 | 0.8 |
| AB Pain Management | 711 (616, 714) | 1.0 | 1.0 | 0.7 |
| GC Hepatobiliary and Pancreatic System Disorders | 1,852 (545, 2,539) | 1.0 | 1.0 | 0.4 |
| JD Skin Disorders | 1,488 (413, 2,237) | 0.9 | 0.9 | 0.8 |

* Maintenance dialysis sessions not included (see Section S1 for further details).

### Table S7: Average number of hospital episodes and hospital cost each year, stratified by time of event occurrence

|  |  | No. of hospital episodes* per 100 person-year | | | |  | Hospital cost (£) per person-year | | | |
| --- | --- | --- | --- | --- | --- | --- | --- | --- | --- | --- |
| Adverse event | N (%) | No history | In year | Previous year | At least  2 years ago |  | No history | In year | Previous year | At least  2 years ago |
| **Vascular event** |  |  |  |  |  |  |  |  |  |  |
| Myocardial infarction | 465 (3.0%) | 3.9 | 11.4 | 8.6 | 6.2 |  | 1489 | 10649 | 4549 | 3400 |
| Coronary revascularisation  (non-urgent) | 292 (1.9%) | 3.9 | 13.1 | 7.8 | 6.1 |  | 1513 | 10972 | 3113 | 2500 |
| Coronary revascularisation  (urgent) | 272 (1.8%) | 3.9 | 13.1 | 7.2 | 5.6 |  | 1510 | 12427 | 3512 | 2966 |
| Transient ischaemic attack | 365 (2.4%) | 3.9 | 9.4 | 6.7 | 6.1 |  | 1519 | 4474 | 3180 | 3222 |
| Ischaemic stroke | 455 (2.9%) | 3.9 | 11.6 | 8.3 | 6.8 |  | 1487 | 10281 | 4376 | 3722 |
| Heart failure | 356 (2.3%) | 3.9 | 14.5 | 11.7 | 10.1 |  | 1483 | 12474 | 7428 | 6677 |
| **Non-vascular event** |  |  |  |  |  |  |  |  |  |  |
| GI tract cancer | 315 (2.0%) | 3.9 | 16.0 | 12.3 | 7.9 |  | 1493 | 12813 | 8231 | 3837 |
| Non-GI tract cancer | 1573 (10.2%) | 3.6 | 14.8 | 12.2 | 7.9 |  | 1336 | 9644 | 6233 | 3597 |
| Intracranial haemorrhage | 100 (0.6%) | 3.9 | 12.0 | 7.6 | 4.9 |  | 1531 | 16138 | 4327 | 2685 |
| GI bleed | 238 (1.5%) | 3.9 | 10.9 | 7.7 | 7.0 |  | 1513 | 9975 | 4626 | 4279 |
| Other major bleed | 117 (0.8%) | 3.9 | 10.4 | 7.7 | 6.4 |  | 1533 | 7254 | 5212 | 3243 |
| Amputation | 143 (0.9%) | 3.8 | 23.9 | 17.5 | 15.4 |  | 1492 | 21745 | 11217 | 8297 |
| End-stage renal disease | 105 (0.7%) | 3.9 | 22.3 | 23.8 | 17.9 |  | 1462 | 25325 | 33603 | 24290 |
| **Deaths** |  |  |  |  |  |  |  |  |  |  |
| Vascular death | 414 (2.7%) | 3.9 | 6.6 | - | - |  | 1525 | 7648 | - | - |
| Vascular event^†^ in year | 191 (1.2%) | 3.9 | 7.7 | - | - |  | 1534 | 9414 | - | - |
| No vascular event in year | 223 (1.4%) | 3.9 | 5.7 | - | - |  | 1538 | 6136 | - | - |
| Non-vascular death | 1126 (7.3%) | 3.9 | 10.5 | - | - |  | 1459 | 10755 | - | - |
| Non-vascular event^‡^ in year | 363 (2.4%) | 3.9 | 11.9 | - | - |  | 1512 | 12734 | - | - |
| No non-vascular event in year | 763 (4.9%) | 3.9 | 9.9 | - | - |  | 1493 | 9813 | - | - |

N (%), no. (percentage) of participants who experienced each adverse event; GI, gastrointestinal. *Hospital episodes refer to hospital inpatient, outpatient and accident and emergency attendances and admissions. Dialysis sessions not included in counts of hospital episodes. See Section S1 for further information. ^†^Vascular event refers to myocardial infarction, coronary revascularisation, transient ischaemic attack, ischaemic stroke, or heart failure. ^‡^ Non-vascular event refers to cancer, intracranial haemorrhage, GI bleed, other major bleed, amputation, or end-stage renal disease.

### Table S8: Annual hospital cost associated with socio-demographic characteristics, clinical risk factors and adverse events for people with diabetes, excluding non-attended outpatient episodes (sensitivity analysis)

| Variable | Annual hospital cost (£) |
| --- | --- |
| (Intercept) | -35 (-70, -1)* |
| Proportion of year observed | 639 (601, 677)** |
| Diabetes type (ref: Type 2) |  |
| Type 1 | 144 (27, 262)* |
| Sex (ref: Male) |  |
| Female | 98 (57, 139)** |
| Townsend index (ref: Q1 (least deprived)) |  |
| Q2 | 10 (-15, 35) |
| Q3 | -2 (-52, 48) |
| Q4 | 162 (91, 233)** |
| Q5 (most deprived) | 509 (337, 681)** |
| Age (per 10 years; centered at 60) |  |
| Age | 93 (69, 118)** |
| Age^2^ | 63 (47, 80)** |
| BMI (ref: <25 kg/m^2^) |  |
| >=25, <30 | 13 (7, 18)** |
| >=30, <35 | 65 (23, 108)** |
| >=35 | 215 (155, 275)** |
| Diabetes duration (ref: <5 years) |  |
| >=5, <10 | 32 (1, 62)* |
| >=10, <20 | 147 (93, 200)** |
| >=20 | 267 (177, 356)** |
| HbA1c (ref: <48 mmol/mol) |  |
| >= 48, < 64 | -5 (-36, 25) |
| >= 64 | 164 (98, 230)** |
| eGFR (ref: >=90 ml/min/1.73m^2^) |  |
| >=60, <90 | 28 (-19, 76) |
| >=45, <60 | 159 (53, 265)** |
| <45 | 643 (410, 875)** |
| Albuminuria (urinary albumin/creatinine ratio >=3 mg/mmol) (ref: N) |  |
| Y | 184 (105, 262)** |
| Retinopathy (ref: N) |  |
| Y | 111 (57, 165)** |
| **Disease history (ref: No history)** |  |
| Myocardial infarction |  |
| In year | 4932 (3662, 6203)** |
| Previous year | 567 (97, 1037)* |
| Coronary revascularizations non-urgent |  |
| In year | 8305 (7466, 9143)** |
| At least 1 year ago | 535 (289, 782)** |
| Coronary revascularizations urgent |  |
| In year |  |
| MI in same year | 4498 (2717, 6279)** |
| No MI in same year | 8366 (5765, 10966)** |
| At least 1 year ago | 111 (-389, 610) |
| Transient ischaemic attack |  |
| In year | 1537 (1105, 1969)** |
| At least 1 year ago | 578 (268, 887)** |
| Ischaemic stroke |  |
| In year | 7126 (6084, 8168)** |
| At least 1 year ago | 1064 (691, 1436)** |
| Heart failure |  |
| In year | 8269 (7146, 9391)** |
| In previous year | 2342 (1694, 2989)** |
| Gastrointestinal tract cancer |  |
| In year | 10165 (8956, 11375)** |
| In previous year | 5134 (3435, 6832)** |
| At least 2 years ago | 1223 (652, 1794)** |
| Other cancers |  |
| In year | 7392 (6884, 7900)** |
| In previous year | 3680 (3205, 4156)** |
| At least 2 years ago | 1597 (1294, 1900)** |
| Intracranial haemorrhage |  |
| In year | 12087 (8858, 15316)** |
| At least 1 year ago | 394 (-91, 880) |
| Gastrointestinal bleed |  |
| In year | 5493 (4370, 6616)** |
| At least 1 year ago | 1195 (591, 1799)** |
| Other major bleeds |  |
| In year | 3871 (2136, 5606)** |
| At least 1 year ago | 675 (214, 1136)** |
| Amputation |  |
| In year | 17719 (15170, 20267)** |
| In previous year | 7146 (5156, 9135)** |
| At least 2 years ago | 3874 (2819, 4928)** |
| End-stage renal disease |  |
| In year | 20897 (18127, 23668)** |
| In previous year | 28829 (25561, 32098)** |
| At least 2 years ago | 20036 (16663, 23408)** |
| **Death in year (ref: No death in year)** | |
| Vascular death |  |
| Vascular event in year | -196 (-1912, 1520) |
| No vascular event in year | 2923 (1728, 4117)** |
| Non-vascular death |  |
| Non-vascular event in year | 2495 (1172, 3819)** |
| No non-vascular event in year | 5956 (5200, 6711)** |

BMI, body mass index; HbA1c, glycated haemoglobin; eGFR, estimated glomerular filtration rate. Vascular event refers to myocardial infarction, coronary revascularisation, transient ischaemic attack, ischaemic stroke, or heart failure. Non-vascular event refers to cancer, intracranial haemorrhage, GI bleed, other major bleed, amputation, or end-stage renal disease.

### Table S9: Self-reported diabetes medication use at baseline and at about 6.5 years into follow-up

|  | At baseline (N = 15436) | At ~ 6.5 years into follow-up (N = 11207) |
| --- | --- | --- |
| GLP-1 receptor agonists | 234 (1.5%) | 513 (3.3%) |
| DPP-4 inhibitors | 347 (2.2%) | 1269 (8.2%) |
| Metformin | 10068 (65.2%) | 7735 (69.0%) |
| Insulin | 3920 (25.4%) | 3062 (27.3%) |
| Sulphonylurea | 4139 (26.8%) | 3387 (30.2%) |

ASCEND participants were asked to report their medication use at baseline and when the EQ-5D-5L (self-rating of health-related quality of life) was administered, which is approximately 6.5 years into follow-up. For the latter time point, medication use was reported by 11207 participants who responded to the EQ-5D-5L questionnaire.

## Supplementary Figures

### Figure S1: Distribution of non-zero annual hospital cost (£)


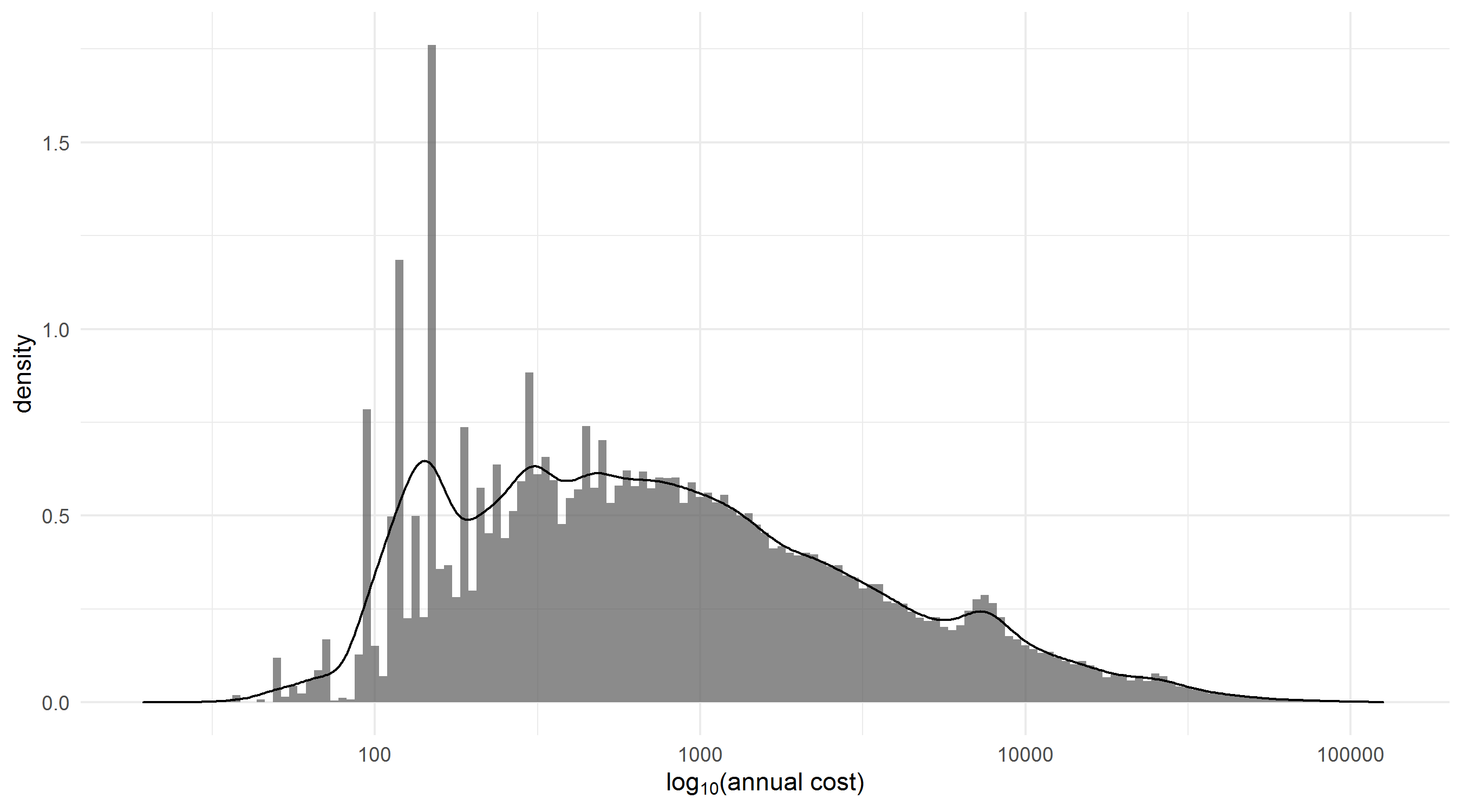


### Figure S2: Marginal effect of age on annual hospital cost


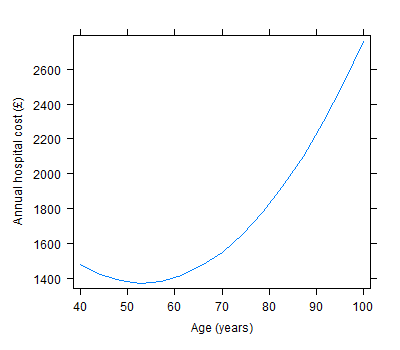


## Section S1: Costing dialysis

HRGs for dialysis are generated from the National Renal Dataset (NDR). Dialysis sessions for chronic kidney diseases are recorded in NDR and healthcare service providers are not expected to record associated admission or attendance in the inpatient or outpatient commissioning dataset (CDS) when patients attend solely for dialysis. Where providers do report dialysis activity within the CDS, the renal HRG LA97A same day dialysis admission or attendance will be generated, which has a 0 cost.^1^

We do not have complete patient-level data on dialysis as we do not have access to NDR. However, dialysis is associated with significant costs and excluding this would severely under-estimate the cost associated with ESRD. Hence, for participants identified to have ESRD, we estimated the cost of dialysis by multiplying the time-on-dialysis with the annual cost of dialysis.

The time-on-dialysis was calculated from date of first report of ESRD to date of transplant, death or censoring. 13 (12%) of the 105 participants identified with ESRD had a transplant recorded within the trial duration, and it was assumed that dialysis was no longer required for these participants post-transplant.

The unit costs of each type of dialysis were obtained from the 2017/18 reference cost^2^ inflated to 2019/20, in line with the main analysis. The average annual dialysis cost is calculated as a weighted average across the different dialysis types, based on the percentage of patients on each dialysis type in 2019 reported by the UK Renal Registry^3^.

#### Table S10: Estimating the average annual cost of dialysis

| Dialysis type | % of prevalent dialysis^3^ | Cost (£) per unit^*^ | Basis of payment | Annual cost (£) |
| --- | --- | --- | --- | --- |
| Haemodialysis (hospital / satellite) | 77.5% | 157 | Per session | 24477^†^ |
| Home haemodialysis | 0.5% | 241 | Per week | 12512 |
| Peritoneal dialysis | 21.9% | 78 | Per day | 28453 |
| **Average annual dialysis cost (£)** | | | | **25283** |

* The unit cost for each dialysis type is the weighted average (based on total no. of sessions recorded in the national schedule of reference cost) across the different dialysis access.^2^ ^†^ Average of 3 haemodialysis sessions per week is assumed.^3^

1. NHS England and NHS Improvement. *2021/22 National Tariff Payment System Annex C: Guidance on Best Practice Tariffs*.; 2021. Accessed February 9, 2022. https://www.england.nhs.uk/wp-content/uploads/2020/11/21-22NT_Annex-C-Best-practice-tariffs.pdf

2. NHS Improvement. *National Schedule of Reference Costs 2017/18*.; 2018.

3. UK Renal Registry. *UK Renal Registry 23rd Annual Report - Data to 31/12/2019*.; 2021. Accessed February 8, 2022. https://ukkidney.org/sites/renal.org/files/publication/file-attachments/23rd_UKRR_ANNUAL_REPORT_0.pdf

## Section S2: Further details on statistical methods

**Missing data imputation**

Less than 2% of participants in ASCEND were missing patient characteristics at baseline. Participants with unknown or “other” ethnicity were grouped together with “White”. Other missing patient characteristics (smoking status, Townsend index, hypertension, retinopathy) were imputed with the modal group (non-smoker, least deprived, with hypertension, no retinopathy, respectively).

About 36% of participants in ASCEND had missing biomarker values as they did not return a usable blood/urine sample at baseline. Missing baseline biomarker values were imputed by multivariate imputation by chained equations using predictive mean matching, with all patient characteristics, clinical risk factors and adverse event occurrence included in the imputation model. This method was selected as it is less sensitive to misspecification of the imputation model, avoids extrapolation beyond the range of data, and retains the distributions of and correlations between variables.^1^ 40 imputations were performed, and the average values across all imputed datasets were used.

The main analysis was performed using observations of annual healthcare costs for each participant (patient-year-level data). Baseline biomarker values were imputed at patient-level using baseline patient characteristics, baseline clinical risk factors, and adverse event occurrence over the follow-up period. The outcome of interest – healthcare cost – is largely driven by occurrences of adverse events which were included in the imputation model, although cost itself was not. Results were similar in sensitivity analysis using only complete cases suggesting that the results are robust to imputation of baseline information.

**Non-linearity of age**

In the single-equation model including all covariates, the martingale residual plot of age suggested that there may be a non-linear relationship between age and annual hospital cost (Figure S3). There was no evidence of non-linearity of age for the two-part model.

A natural cubic spline was fitted to explore the shape of the relationship. The shape of the spline fitted suggested that the polynomial form with degree 2 might be an appropriate functional form for age (Figure S4). The model with the polynomial form of age has MSE but higher MAPE than the model with the spline form of age (Table S11). Despite so, the marginal effect of the polynomial form of age matches that of the natural cubic spline (Table S12) moderately well. Hence the polynomial form was selected for easier computation.

| Figure S3: Martingale residual plot | Figure S4: Marginal effect of age (natural cubic spline) |
| --- | --- |
| 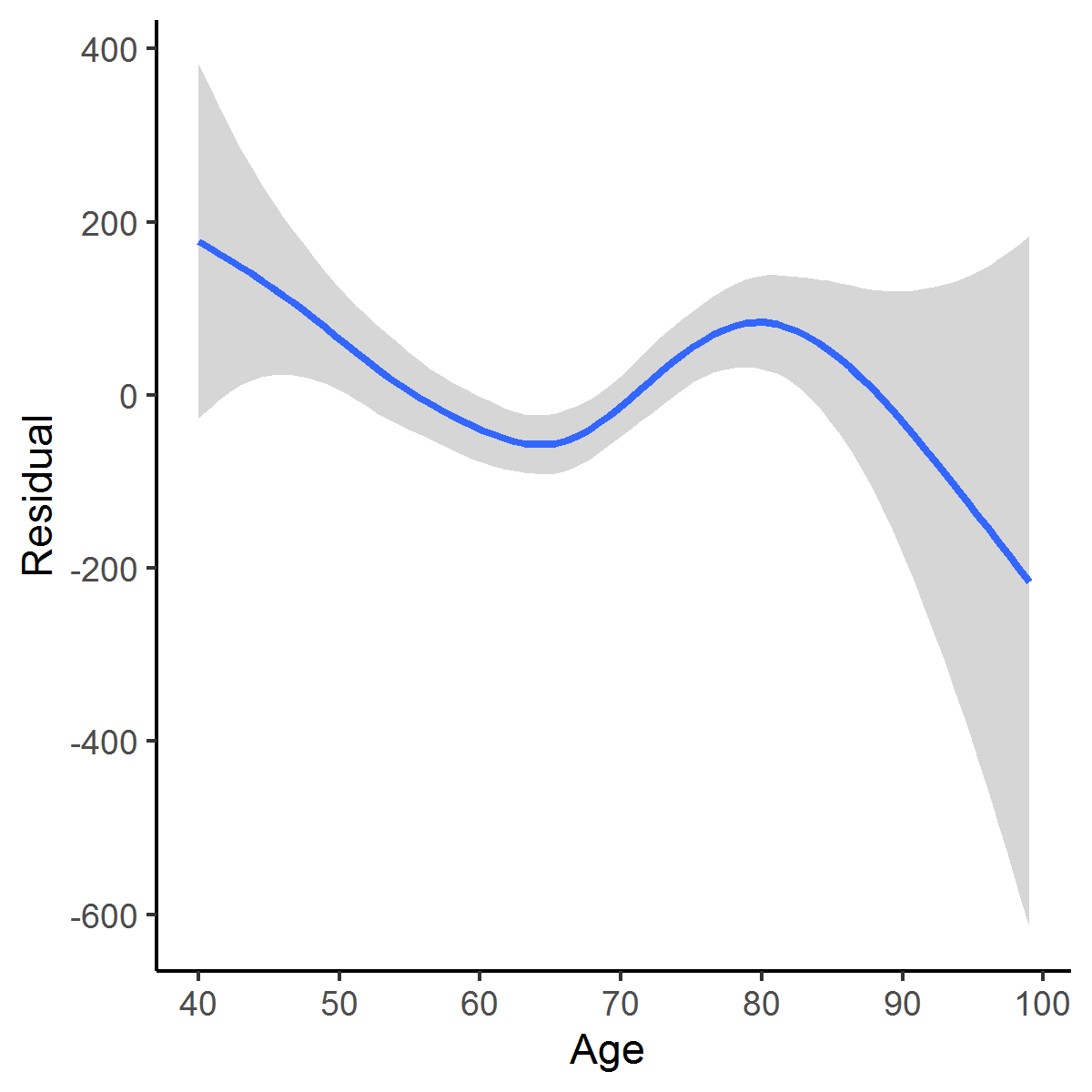 | 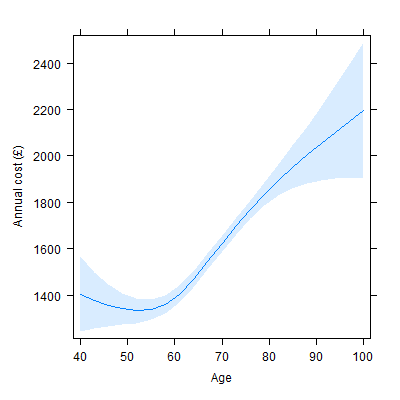 |

#### Table S11: Predictive performance of models using different forms of age

| Form of age | MSE | RMSE | MAPE | MPE | R^2^ |
| --- | --- | --- | --- | --- | --- |
| Linear | 13935498 | 3733 | 1610 | 0 | 0.233 |
| Natural cubic spline | 13963995 | 3737 | 1581 | -68.4 | 0.234 |
| Polynomial degree 2 | 13938125 | 3733 | 1625 | 21.4 | 0.233 |

MSE: mean squared error; RMSE: root mean squared error; MAPE: mean absolute prediction error; MPE: mean prediction error; R^2^: Efron’s R^2^, square of the correlation between the predicted and observed values

#### Table S12: Marginal effects of age on annual hospital cost (£)

|  | Marginal effect of age relative to 60 years old | | | | |
| --- | --- | --- | --- | --- | --- |
| Form of age | 50 | 60 | 70 | 80 | 90 |
| Linear | -122 | 0 | 130 | 256 | 382 |
| Natural cubic spline | -17 | 0 | 128 | 353 | 643 |
| Polynomial degree 2 | -22 | 0 | 159 | 445 | 860 |

**Model selection**

Given the features of the annual hospital costs data, we considered both single-equation models and two-part models, with the first part predicting the likelihood of hospital use (thus incurring non-zero cost) and the second part predicting the cost conditional on hospital use. Logistic regression was used to estimate the likelihood of hospital use for the first part of the two-part model. Generalised linear models (GLMs) with the identity, log and square-root link functions were considered for the single-equation model and for the second part of the two-part model, and the Gaussian, Poisson and Gamma distributions were considered for modelling the mean-variance relationship in the data.

The appropriateness of each link function was assessed using the Pregibon link test^2^ and the modified Hosmer-Lemeshow test^3^ (Table S13). We find the tests generally rejected the log link. The modified Park test^4^ indicated that the Poisson and Gamma distribution may be appropriate for the identity and square-root links. The Copas test implemented using 3-fold cross validation repeated 1000 times was used to assess out-of-sample performance of the models.^5^ The identity link had the lowest rejection rate, and overfitting was evident for models with the log link.

Finally, the performance of the models was evaluated based on mean prediction error (MPE), mean absolute prediction error (MAPE), root-mean-squared error (RMSE), and Efron’s 𝑅^2^ (Table S13). Generally, models with the identity link demonstrated the best performance with low bias, and displayed no systematic bias across deciles of predicted cost (Table S14). Plots of the MPE by decile (Figure S4) indicated that models with log link have significant bias in the highest decile of predicted costs. This bias was worst for the Gamma variance.

Overall, from the assessments above, the GLM with Poisson-identity specification has the best overall performance for both the single-equation and two-part models. Generally, the single equation models performed similarly to two-part models. The marginal impact on annual hospital costs associated with adverse events estimated by the single-equation and the two-part models were similar (Table S15). Hence, the single-equation GLM with Poisson-identity specification was chosen for parsimony.

#### Table S13: Predictive performance of candidate models

|  |  | Single-equation models | | | | |  | Two-part models | | | | |
| --- | --- | --- | --- | --- | --- | --- | --- | --- | --- | --- | --- | --- |
| Link | Distribution | MSE | RMSE | MAPE | MPE | R^2^ |  | MSE | RMSE | MAPE | MPE | R^2^ |
| Identity | Gaussian | 14184050 | 3766 | 1622 | 0.00 | 0.301 |  | 14189530 | 3767 | 1614 | 1.52 | 0.301 |
| Identity | Poisson | 14310384 | 3783 | 1606 | -58.90 | 0.299 |  | 14216319 | 3770 | 1614 | 1.56 | 0.299 |
| Identity | Gamma | 14245401 | 3774 | 1605 | -22.86 | 0.298 |  | 14238227 | 3773 | 1612 | -1.26 | 0.298 |
| Log | Gaussian | 15500057 | 3937 | 1894 | 243.86 | 0.242 |  | 15088376 | 3884 | 1801 | 158.84 | 0.259 |
| Log | Poisson | 20226215 | 4497 | 1711 | 0.00 | 0.160 |  | 18096108 | 4254 | 1685 | 5.25 | 0.191 |
| Log | Gamma | 868440661 | 29469 | 2178 | 599.43 | 0.037 |  | 128919260 | 11354 | 1853 | 256.24 | 0.077 |
| Square-root | Gaussian | 14405148 | 3795 | 1799 | 280.76 | 0.294 |  | 14271304 | 3778 | 1660 | 57.71 | 0.297 |
| Square-root | Poisson | 14452626 | 3802 | 1617 | 0.00 | 0.291 |  | 14429045 | 3799 | 1619 | 3.18 | 0.291 |
| Square-root | Gamma | 14740115 | 3839 | 1629 | 33.67 | 0.285 |  | 14662875 | 3829 | 1627 | 29.20 | 0.286 |

MSE: mean squared error; RMSE: root mean squared error; MAPE: mean absolute prediction error; MPE: mean prediction error; R^2^: Efron’s R^2^, square of the correlation between the predicted and observed values

#### Figure S4: Mean prediction error of candidate models in each decile of predicted cost

**
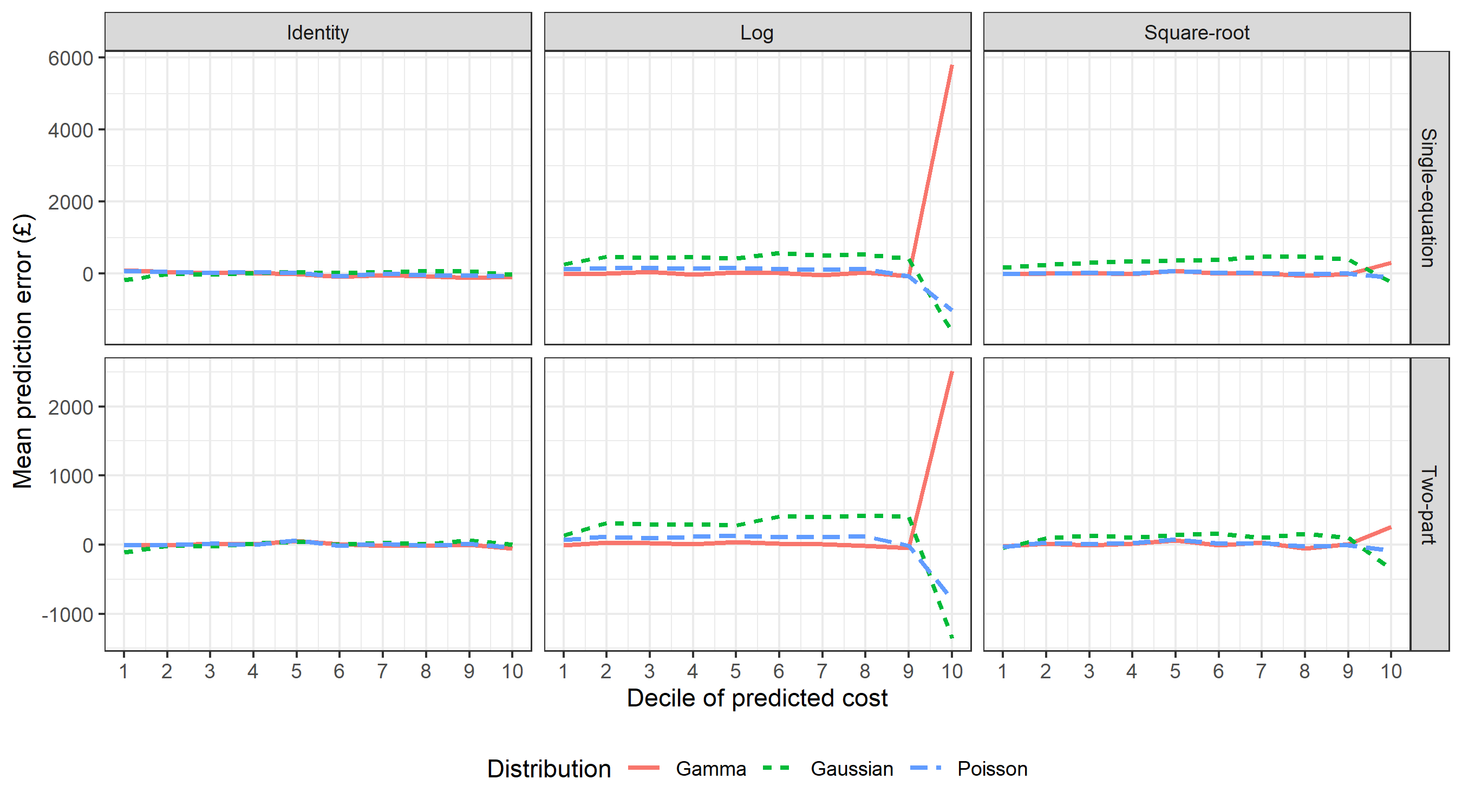
**

#### Table S14: Mean prediction error of models with identity link in each decile of predicted cost

|  | Single-equation models | | |  | Two-part models | | |
| --- | --- | --- | --- | --- | --- | --- | --- |
| Decile | Gaussian | Poisson | Gamma |  | Gaussian | Poisson | Gamma |
| 1 | -184.1 | 81.5 | 92.5 |  | -109.5 | -6.9 | -2.6 |
| 2 | -10.5 | 54.9 | 49.4 |  | -16.2 | -7.9 | -5.8 |
| 3 | -24.4 | 23.2 | 12.1 |  | -19.9 | 17.4 | 17.6 |
| 4 | 22.6 | 50.0 | 25.5 |  | 11.9 | 1.4 | 9.3 |
| 5 | 39.3 | 17.3 | -6.9 |  | 47.0 | 64.6 | 54.1 |
| 6 | 16.5 | -66.4 | -91.4 |  | -2.0 | -19.1 | 7.9 |
| 7 | 35.7 | -1.1 | -47.6 |  | 26.9 | 18.7 | -16.4 |
| 8 | 63.4 | -38.7 | -66.6 |  | 8.0 | -16.4 | -18.6 |
| 9 | 65.3 | -49.8 | -119.2 |  | 66.1 | 11.9 | -4.2 |
| 10 | -23.8 | -71.0 | -86.3 |  | 2.9 | -48.0 | -53.9 |

#### Table S15: Excess annual hospital cost associated with adverse event occurrence estimated by the single equation model and the two-part model

| Disease history (ref: no event) | Excess annual hospital costs (£, 95% CI) | |
| --- | --- | --- |
|  | Single-equation model | Two-part model |
| Myocardial infarction |  |  |
| In year | 4114 (3016, 5212) | 3832 (2947, 4830) |
| In previous year | 1162 (277, 2047) | 1204 (505, 1884) |
| At least 2 years ago | 268 (-180, 715) | 299 (-80, 721) |
| Coronary revascularisation (non-urgent) |  |  |
| In year | 8388 (7549, 9226) | 8319 (7440, 9154) |
| In previous year | 741 (258, 1225) | 786 (351, 1406) |
| At least 2 years ago | 432 (164, 699) | 503 (242, 812) |
| Coronary revascularisations (urgent) |  |  |
| In year | 5734 (4311, 7158) | 5201 (4014, 6438) |
| In previous year | 118 (-859, 1095) | 77 (-426, 769) |
| At least 2 years ago | 146 (-332, 624) | 188 (-232, 668) |
| Transient ischaemic attack |  |  |
| In year | 1582 (1151, 2013) | 1526 (1148, 1966) |
| In previous year | 466 (43, 889) | 475 (116, 942) |
| At least 2 years ago | 631 (275, 986) | 685 (339, 1113) |
| Ischaemic stroke |  |  |
| In year | 6857 (5815, 7899) | 6661 (5699, 7728) |
| In previous year | 1348 (666, 2029) | 1302 (781, 2019) |
| At least 2 years ago | 1002 (594, 1411) | 1023 (617, 1467) |
| Heart failure |  |  |
| In year | 7935 (6842, 9028) | 7638 (6602, 8745) |
| In previous year | 3180 (2168, 4192) | 3035 (2071, 4145) |
| At least 2 years ago | 1826 (1107, 2546) | 1786 (1065, 2637) |
| GI tract cancer |  |  |
| In year | 9879 (8673, 11084) | 9697 (8584, 10935) |
| In previous year | 5165 (3460, 6871) | 5121 (3656, 6955) |
| At least 2 years ago | 1186 (618, 1754) | 1223 (694, 1943) |
| Other non-GI tract cancers |  |  |
| In year | 7098 (6608, 7588) | 6978 (6546, 7440) |
| In previous year | 3719 (3240, 4198) | 3673 (3189, 4199) |
| At least 2 years ago | 1596 (1292, 1900) | 1639 (1336, 1969) |
| Intracranial haemorrhage |  |  |
| In year | 11816 (8519, 15113) | 11542 (8638, 14596) |
| In previous year | 995 (84, 1906) | 1005 (90, 2003) |
| At least 2 years ago | 287 (-268, 843) | 293 (-190, 995) |
| GI bleed |  |  |
| In year | 5293 (4153, 6432) | 5156 (4078, 6362) |
| In previous year | 1605 (728, 2481) | 1556 (774, 2531) |
| At least 2 years ago | 1060 (431, 1688) | 1134 (511, 1900) |
| Other major bleed |  |  |
| In year | 3906 (2159, 5653) | 3817 (2379, 5634) |
| In previous year | 1139 (158, 2121) | 1199 (293, 2347) |
| At least 2 years ago | 522 (59, 985) | 557 (63, 1102) |
| Amputation |  |  |
| In year | 17656 (15096, 20216) | 17246 (14740, 19875) |
| In previous year | 7061 (5025, 9097) | 6898 (4903, 9190) |
| At least 2 years ago | 3858 (2786, 4930) | 3713 (2638, 4829) |
| End-stage renal disease |  |  |
| In year | 20758 (17942, 23574) | 20785 (17961, 23951) |
| In previous year | 28821 (25511, 32131) | 28871 (25492, 32280) |
| At least 2 years ago | 20042 (16693, 23391) | 20463 (16971, 23854) |
| **Death in year (ref: No death in year)** | | |
| Vascular death | 2155 (1162, 3148) | 1612 (901, 2385) |
| Non-vascular death | 5394 (4732, 6055) | 4272 (3740, 4853) |

GI, gastrointestinal. Other major bleed refers to bleeding events that are not intracranial haemorrhage or GI bleed. Model does not include interaction terms. Estimation adjusted for other socio-demographic and clinical risk factors.

*Average marginal effect for two-part model estimated using 1000 bootstrap.^6^

**References**

1. Roderick J. A. Little. “Missing-Data Adjustments in Large Surveys.” *Journal of Business & Economic Statistics* 6, no. 3 (1988): 287–96. doi:10.2307/1391878
2. Pregibon D. Goodness of Link Tests for Generalized Linear Models. *J R Stat Soc Ser C Appl Stat*. 1980;29(1):15-14. doi:10.2307/2346405
3. Hosmer DW, Lemeshow S, Klar J. Goodness-of-Fit Testing for the Logistic Regression Model when the Estimated Probabilities are Small. *Biom J*. 1988;30(8):911-924. doi:10.1002/bimj.4710300805
4. Park RE. Estimation with Heteroscedastic Error Terms. *Econometrica*. 1966;34(4):888-888. doi:10.2307/1910108
5. Jones AM. *Models for Health Care*. Oxford University Press; 2011. doi:10.1093/oxfordhb/9780195398649.013.0024
6. Li Z, Mahendra G. Using “Recycled Predictions” for Computing Marginal Effects. In: *SAS Global Forum 2010*. ; 2010.
